# Intra-Arterial Thrombolysis Vs. Mechanical Thrombectomy in Acute Minor Ischemic Stroke Due to Large Vessel Occlusion

**DOI:** 10.3389/fneur.2022.860987

**Published:** 2022-07-12

**Authors:** Dapeng Sun, Xiaochuan Huo, Raynald, Anxin Wang, Dapeng Mo, Feng Gao, Ning Ma, Zhongrong Miao

**Affiliations:** ^1^Department of Interventional Neuroradiology, Beijing Tiantan Hospital, Capital Medical University, Beijing, China; ^2^China National Clinical Research Center for Neurological Diseases, Beijing, China

**Keywords:** minor ischemic stroke, large vessel occlusion, outcome, mechanical thrombectomy, intra-arterial thrombolysis

## Abstract

**Background:**

The efficacy and safety of mechanical thrombectomy (MT) in acute large vessel occlusion (LVO) patients with minor stroke (NIHSS ≤ 5) remains undetermined. We aimed to compare the efficacy and safety of intra-arterial thrombolysis (IAT) alone vs. MT for LVO patients with minor stroke.

**Methods:**

Patients were selected from the Acute Ischemic Stroke Cooperation Group of Endovascular Treatment (ANGEL) registry, a prospective multicenter registry study, and divided into MT and IAT alone groups. We compared the outcome measures between the two groups, including 90-day functional outcome evaluated by the modified Rankin Scale (mRS), the final recanalization level, intracranial hemorrhage, and mortality within 90-days by logistic regression models with adjustment. Besides the conventional multivariable analysis, we performed a sensitivity analysis by adjusting the propensity score to confirm our results. The propensity score was derived using a logistic regression model.

**Results:**

Of the 120 patients, 63 received IAT alone and 57 received MT as the first-line treatment strategy. As compared to MT group, patients in the IAT alone group were associated with a higher chance of 90-day mRS 0-2 [93.7% vs. 71.9%, odds ratio (OR) = 4.75, 95% confidence interval (CI): 1.20–18.80, *P* = 0.027], a high chance of 90-day mRS 0-3 (96.8% vs. 86.7%, OR = 11.35, 95% CI: 1.93–66.86, *P* = 0.007), a shorter median time from puncture to recanalization (PTR) (60 min vs. 100 min, β = −63.70, 95% CI: −81.79– −45.61, *P* < 0.001), a lower chance of any intracranial hemorrhage (ICH) within 48 h (3.2% vs. 19.3%, OR = 0.15, 95% CI: 0.03–0.79, *P* = 0.025), and a lower chance of mortablity within 90 days (1.6% vs. 9.2%, OR = 0.05, 95% CI: 0.01–0.57, *P* = 0.016). Similarly, the sensitivity analysis showed the robustness of the primary analysis.

**Conclusions:**

Compared with MT, IAT may improve 90-day clinical outcomes with decreased ICH rate and mortality in LVO patients with minor stroke.

## Introduction

Minor stroke is not uncommon, occuring in about two-thirds of the entire acute ischemic stroke (AIS) population (1). An underlying large vessel occlusion (LVO) may increase the risk of further clinical deterioration in this population (2). Several well-known randomized controlled trials (RCT) have demonstrated the clinical benefit of mechanical thrombectomy (MT) over standard medical care in AIS patients with large vessel occlusion (3). Confirming this, the international stroke guidelines (4, 5) have also provided a high level of evidence and treatment recommendations. However, a majority of these trials enrolled patients with baseline National Institutes of Health Stroke Scale (NIHSS) scores of >5 (3, 6), and therefore the efficacy and safety of MT for LVO patients with a minor stroke (NIHSS ≤ 5) remain unclear.

Previous studies report that, compared to standard medical treatment, MT could result in similar clinical outcomes and a higher risk of intracranial hemorrhage (ICH) in LVO patients with minor stroke (7, 8). Unlike MT, which has an invasive characteristic concerning arterial wall damage, non-MT treatment, such as intra-arterial thrombolysis (IAT), seems to be an appropriate treatment option for LVO with minor stroke. Additionally, IAT had a better rate of recanalization when compared with standard medical treatment (9) and could also enhance the microcirculatory reperfusion of the target occlusion artery (10).

Therefore, we sought to compare the efficacy and safety of IAT alone vs. MT for acute LVO patients with minor stroke from a multicenter prospective registry in China.

## Methods

### Study Population

Patients from the Acute Ischemic Stroke Cooperation Group of Endovascular Treatment (ANGEL) registry, a multicenter, prospective registry study from June 2015 to December 2017, were included in the data analysis (11). They were screened further to meet the following criteria: (1) age more than 18 years; (2) clinical diagnosis of ischemic stroke in which the stroke symptoms lasted for more than 30 min and showed no improvement before treatment; (3) patients with minor stroke (NIHSS 0-5); (4) patients with large vessel occlusion confirmed by digital subtraction angiography (DSA) including the internal carotid artery, the middle cerebral artery (M1/2/3 segment), the anterior cerebral artery, the basilar artery, or the dominant vertebral artery, and the posterior cerebral artery; and (5) patients who underwent IAT alone or MT.

### Data Collection

We prospectively collected all the variables, including (1) clinical: age, sex, vascular risk factors, medical history, baseline NIHSS, procedure details, periprocedural management, the time points of working flow, and functional outcomes [e.g., modified Rankin scale (mRS)] and (2) imaging: baseline CT/ magnetic resonance (MR) and CT angiography/MR angiography imaging, DSA, and postprocedural CT.

The imaging data were evaluated by the imaging core laboratory consisting of three trained and experienced neuroradiologists blinded to the clinical data and outcomes. Two neuroradiologists reviewed all imaging independently, with a third available for adjudication when needed.

The imaging core laboratory assessed the Alberta Stroke Program Early CT Score (ASPECTS) on baseline CT (12), modified Thrombolysis in Cerebral Infarction (mTICI) (13), procedure-related complications (e.g., intraprocedural embolization, arterial perforation, arterial dissection, and vasospasm requiring treatment) on DSA, and intracranial hemorrhage (ICH) after EVT on postprocedural CT.

### Endovascular Treatment

Patients from the MT group underwent MT (stent retriever or/and contact aspiration) as the first-line endovascular treatment strategy. The rescue treatment, such as balloon angioplasty or stenting, was allowed at the surgeon's discretion. Patients from IAT alone group received only IAT as the sole endovascular treatment.

IAT can be conducted on patients from the ANGEL registry based on the surgeon's discretion. According to PROACT-II (Prolyse in Acute Cerebral Thromboembolism II) (9) and MELT (Middle Cerebral Artery Embolism Local Fibrinolytic Intervention Trial) (14) studies, we performed intra-arterial thrombolysis before or next to and distal to the thrombus by injecting urokinase (UK) or recombinant tissue plasminogen (r-tPA) manually *via* the microcatheter. The best dose and rate were not fixed, and we suggested 1 mg/min r-tPA for no more than 40 mg or intra-arterial (IA) 10–30 thousand unit/min urokinase for no more than 1 million units. If the patients had received intravenous r-tPA previously, we suggested an intra-arterial dosage of <30 mg alteplase or 400,000 U urokinase (11).

### Outcome Measurement

We assessed 90-day functional outcomes using the mRS by a standardized telephone interview performed by trained investigators blinded to clinical information. The primary outcome was 90-day mRS 0-2. The secondary outcomes were 90-day mRS 0-1, 90-day mRS 0-3, time from puncture to recanalization (PTR), time from onset to recanalization (OTR), successful recanalization, and complete recanalization. The safety outcomes were any ICH within 48 h and mortality (mRS 6) within 90 days. Successful recanalization was defined as mTICI 2b-3 at the end of the procedure, and complete recanalization was defined as mTICI 3 at the end of the procedure.

### Statistical Analysis

Categorical variables were expressed as numbers (percentage), and continuous variables were expressed as median (interquartile range [IQR]). We performed an univariable analysis using the Mann-Whitney *U*-test for continuous variables and χ^2^ or Fisher's exact test for categorical variables to identify the difference between the IAT alone and MT groups. For comparing the outcome measures, all the significant baseline characteristics in the univariate analysis (*P* < 0.05) and the baseline variables likely to influence clinical outcomes as potential confounders (age, sex, NIHSS, bridging IVT, tirofiban during the procedure, heparin during the procedure, and occlusion location) were adjusted by binary logistic regression model or generalized linear model as appropriate to analyze the adjusted odds ratios (OR) or β-coefficients with their 95% confidence intervals (CI).

In addition to conventional multivariable analysis, we performed a sensitivity analysis by adjusting the propensity score, derived using a logistic regression model that included all the potential confounders above. A two-sided *P*-value of < 0.05 was considered to be statistically significant. SPSS version 26.0 (IBM, Armonk, NY, USA) was used to analyze the data.

## Results

**Figure 1:**
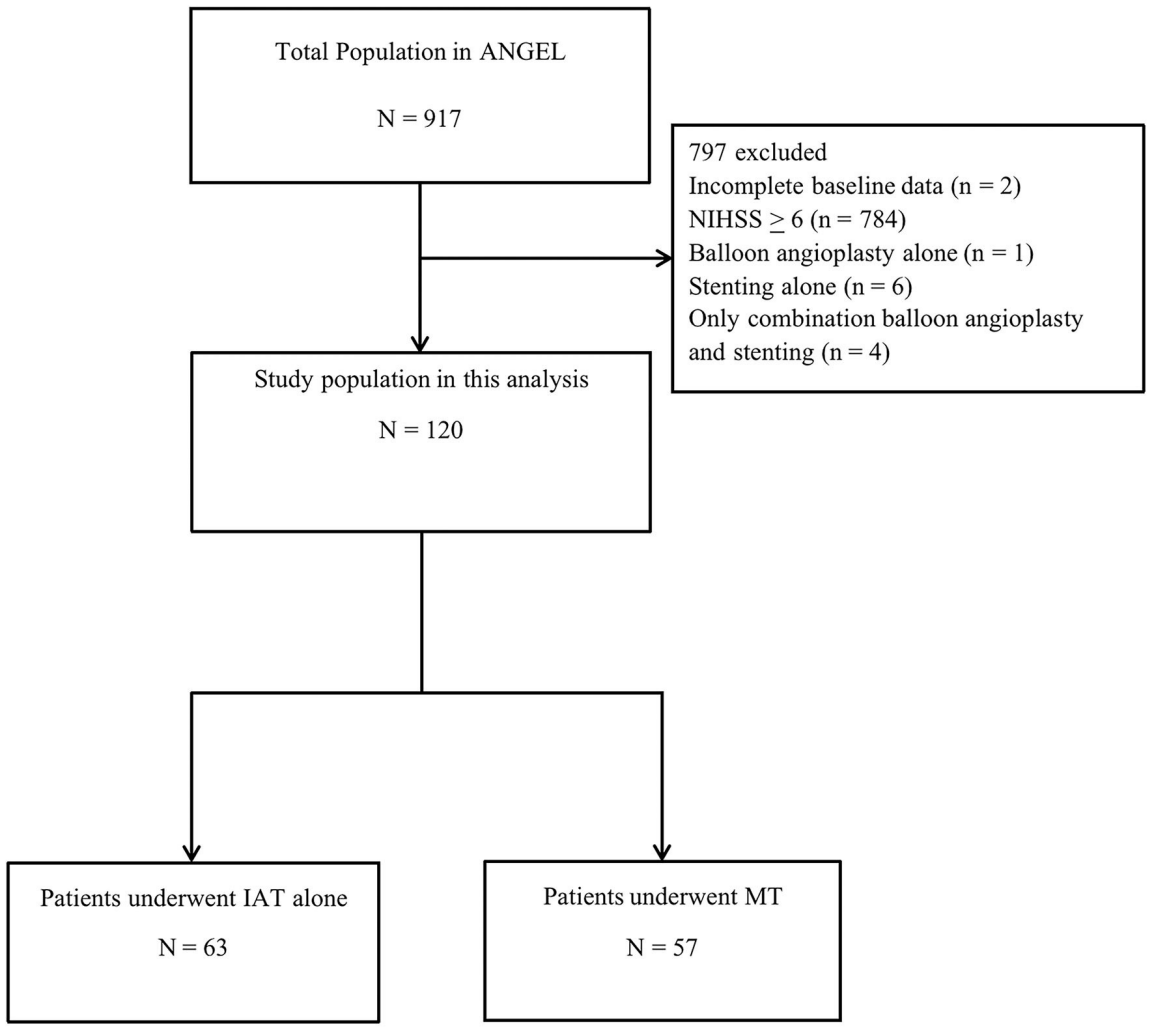
Flow chart showing patient selection. MT, mechanical thrombectomy; IAT, intra-arterial thrombolysis; NIHSS, National Institutes of Health Stroke Scale score.

As shown in Figure 1, 797 of the 917 patients were excluded for the following reasons: (1) incomplete baseline data (*n* =2); (2) NIHSS ≥ 6 (*n* = 784); (3) stenting alone (*n* = 6); (4) balloon angioplasty alone (*n* = 1); and (4) only stenting and balloon angiography (*n* = 4). Finally, our study included 120 patients. Of the 120 patients, 63 were in the IAT alone group and 57 were in the MT group.

### Baseline Characteristics

Table 1 shows the baseline characteristics according to the MT and IAT alone group. Patients in the IAT alone group were younger than the MT group [60(52–65) vs. 65(59–75), *P* = 0.001]. Patients in the IAT alone group had a lower rate of bridging IVT (6.3% vs. 24.6%, *P* = 0.005), a lower dose of tirofiban during EVT (25.4% vs. 49.1%, *P* = 0.007), and a lower dose of heparin during EVT (17.5% vs. 38.6%, *P* = 0.010) than patients in the MT group.

**Table 1:** Baseline characteristics of IAT alone and MT group in LVO patients with minor stroke.

**Characteristics**	**Total (*n* = 120)**	**IAT alone (*n* = 63)**	**MT (*n* = 57)**	***P-*value**
Age–year, median (IQR)	62 (53–70)	60 (52–65)	65 (59–75)	**0.001**
Male sex–no (%)	89 (74.2)	45 (71.4)	44 (77.2)	0.471
SBP–mmHg, median (IQR)	145 (133–160)	140 (135–160)	148 (130–166)	0.613
ASPECTS, median (IQR)	8 (7–8)	8 (7–8)	8 (7–8)	0.174
NIHSS score, median (IQR)	3 (1–4)	3 (1–4)	3 (1–4)	0.829
Current or previous smoking–no (%)	46 (38.3)	24 (38.1)	22 (38.6)	0.955
Current or previous drinking–no (%)	27 (22.5)	14 (22.2)	13 (22.8)	0.939
**Medical history–no (%)**				
Atrial Fibrillation	4 (3.3)	1 (1.6)	3 (1.9)	0.345
Diabetes Mellitus	26 (21.7)	10 (15.9)	16 (28.1)	0.105
Previous stroke	8 (6.7)	2 (3.2)	6 (10.5)	0.148
Hypertension	68 (56.7)	32 (50.8)	36 (63.2)	0.172
**TOAST classification–no (%)**				
Large artery atherosclerosis	102 (85.0)	56 (88.9)	46 (80.7)	0.210
Cardiogenic	3 (2.5)	0 (0)	3 (5.3)	0.104
Other etiology or unknown etiology	15 (12.5)	7 (11.1)	8 (14.0)	0.692
**Occlusion site confirmed by DSA–no (%)**				0.238
ICA	36 (30.0)	17 (27.0)	19 (33.3)	
M1	19 (15.8)	12 (19.1)	7 (12.3)	
M2/3	7 (5.8)	4 (6.3)	3 (5.3)	
ACA	3 (2.5)	2 (3.2)	1 (1.8)	
PCA	8 (6.7)	7 (11.1)	1 (1.8)	
V-BA	47 (39.2)	21 (33.3)	26 (45.6)	
Anterior circulation–no (%)	65 (54.2)	35 (55.6)	30 (52.6)	0.748
Posterior circulation–no (%)	55 (45.8)	28 (44.4)	27 (47.4)	
OTD time, median (IQR), min	264 (180–328)	281 (180–360)	250 (153–301)	0.198
DTP time, median (IQR), min	131 (109–200)	130 (92–199)	131 (119–203)	0.130
OTP time, median (IQR), min	430 (343–550)	443 (330–670)	430 (360–493)	0.499
**Peri-procedural antithrombotic and anticoagulant–no (%)**				
Prior use of antiplatelet agents	29 (24.2)	13 (20.6)	16 (28.1)	0.342
Bridging IVT	18 (15.0)	4 (6.3)	14 (24.6)	**0.005**
Tirofiban during the procedure	44 (36.7)	16 (25.4)	28 (49.1)	**0.007**
Heparin during the procedure	33 (27.5)	11 (17.5)	22 (38.6)	**0.010**

*MT, mechanical thrombectomy; SD, standard deviation; SBP, systolic blood pressure; IQR, interquartile range; NIHSS, National Institutes of Health Stroke Scale score; ASPECTS, Alberta Stroke Program Early CT score, TOAST, trial of ORG 10172 in acute stroke treatment; ICA, internal carotid artery; M1, middle cerebral artery M1 segment; M2/3, middle cerebral artery M2/3 segment; ACA, anterior cerebral artery; PCA, posterior cerebral artery; V-BA, vertebrobasilar artery; OTD, time from onset to door; DTP, time from door to puncture; PTR, time from puncture to recanalization; OTP, time from onset to puncture; OTR, time from onset to recanalization; IVT, intravenous thrombolysis; IAT, intra-arterial thrombolysis. Bold values indicates statistical significance*.

### Outcome Measures

**Figure 2:**
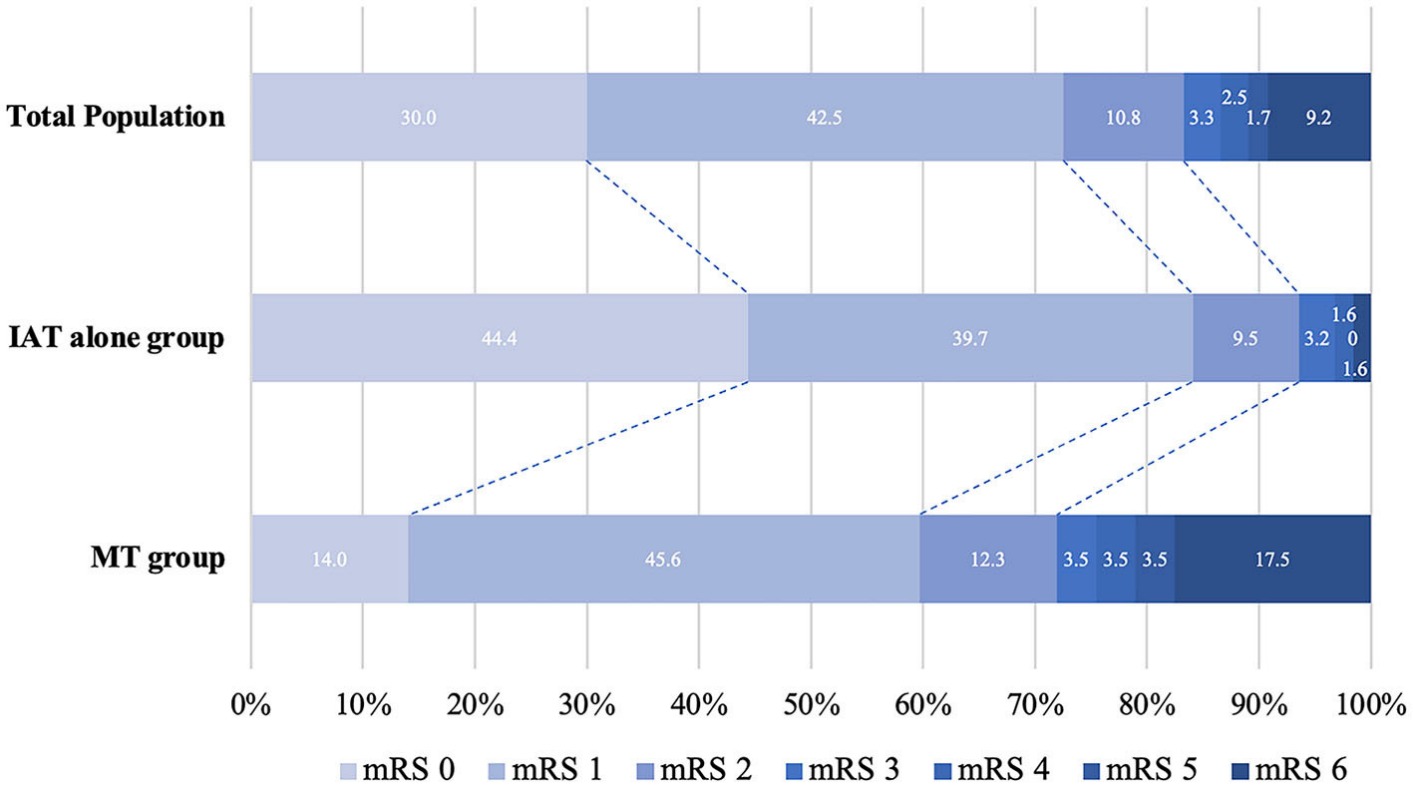
Distribution of modified Rankin Scale (mRS) scores at 3 months between IAT alone and MT group. MT, mechanical thrombectomy; IAT, intra-arterial thrombolysis; NIHSS, National Institutes of Health Stroke Scale score; mRS, modified Rankin scale.

Table 2 shows the comparison of the outcome measures between the IAT alone and MT groups. Regarding the primary outcome (90-day mRS 0-2), patients in the IAT alone group had a higher 90-day mRS 0-2 rate than patients in the MT group (93.7% vs. 71.9%, OR = 4.75, 95% CI: 1.20–18.80, *P* = 0.027) (Figure 2). Regarding the secondary outcomes, the IAT alone group had higher rates of 90-day mRS 0-3 (96.8% vs. 75.4%, OR = 11.35, 95% CI: 1.93–66.86, *P* = 0.007) and shorter PTR [60(40–80) min vs. 100(80–157) min, β = −63.70, 95% CI: −81.79– −45.61, *P* < 0.001] than the MT group. Regarding the safety outcomes, the IAT alone group had less ICH within 48 h (3.2% vs. 19.3%, OR = 0.15, 95% CI: 0.03–0.79, *P* = 0.025) and mortality (mRS 6) within 90 days (1.6% vs. 17.5%, OR = 0.05, 95% CI: 0.01–0.57, *P* = 0.016). However, the angiographic outcomes (successful recanalization and complete recanalization) were similar between the two groups (all *P* > 0.05).

**Table 2:** Comparison of outcomes between IAT alone and MT groups.

**Variables**	**Overall population** ***n*** **=** **120**			**Unadjusted model**		**Adjusted model^a^**	
	**Total (*n* = 120)**	**IAT alone** **(*n* = 63)**	**MT (*n* = 57)**	**Effect size** **(95% CI)**	***P*–value**	**Effect size** **(95% CI^a^)**	***P-*value**
**Primary outcome-no (%)**							
90-day mRS 0-2	100 (83.3)	59 (93.7)	41 (71.9)	5.76 (1.79–18.47)	0.003	4.75 (1.20–18.80)	**0.027**
**Secondary outcomes**							
90-day mRS 0-1- no (%)	87 (72.5)	53 (84.1)	34 (59.6)	3.59 (1.52–8.46)	0.004	2.44 (0.88–6.71)	0.085
90-day mRS 0-3- no (%)	104 (86.7)	61 (96.8)	43 (75.4)	9.93 (2.15–45.96)	0.003	11.35 (1.93–66.86)	**0.007**
Successful recanalization-no (%)	108 (90.0)	57 (90.5)	51 (89.5)	1.12 (0.34–3.69)	0.855	0.29 (0.06–1.49)	0.285
Complete recanalization-no (%)	87 (72.5)	47 (74.6)	40 (70.2)	1.25 (0.56–2.79)	0.588	0.73 (0.28–1.91)	0.517
PTR, median (IQR), min	80 (50–110)	60 (40–80)	100 (80–157)	−50.18 (−66.89–33.48)^b^	<0.001	−63.70 (−81.79–−45.61)^b^	<0.001
OTR, median (IQR), min	514 (433–664)	510 (395–730)	528 (450–648)	3.48 (−84.03–90.99)^b^	0.938	−11.90 (−104.09–80.30)^b^	0.800
**Safety outcomes-no (%)**							
Any ICH within 48 h	13 (10.8)	2 (3.2)	11 (19.3)	0.14 (0.03–0.65)	0.012	0.15 (0.03–0.79)	**0.025**
Mortality within 90 days (mRS 6)	11 (9.2)	1 (1.6)	10 (17.5)	0.08 (0.01–0.61)	0.016	0.05 (0.01–0.57)	**0.016**

*ICH, intracranial hemorrhage; mTICI, modified thrombolysis in cerebral infarction; mRS, modified Rankin scale; IAT, intra-arterial thrombolysis; MT, mechanical thrombectomy; PTR, time from puncture to recanalization; OTR, time from onset to recanalization; OR, odds ratio; CI, confidence interval*.

a *Adjusted for age, sex, NIHSS, intravenous thrombolysis, tirofiban and heparin use during the procedure, and occlusion location*.

b *The β-coefficients were calculated using a generalized linear model*.

*Bold values indicates statistical significance*.

### Sensitivity Analysis

After the sensitivity analysis, we also found that the IAT alone group was independently associated with a higher chance of 90-day mRS 0-2 (OR = 4.17, 95% CI: 1.17–14.89, *P* = 0.028) and 90-day mRS 0-3 (OR = 9.79, 95% CI: 1.89–50.60, *P* = 0.006), a lower chance of any ICH within 48 h (OR = 0.13, 95% CI: 0.03–0.71, *P* = 0.019) and mortality within 90 days (OR = 0.06, 95% CI: 0.01–0.52, *P* = 0.011), and shorter PTR (β = −61.44, 95% CI: −80.05– −42.82, *P* < 0.001) as compared with the MT group (Table 3).

**Table 3:** Sensitivity analysis: comparison of outcomes between IAT alone and MT groups with adjustment for the propensity score.

**Variables**	**Overall population** ***n*** **=** **120**			**Adjusted model^a^**	
	**Total (*n* = 120)**	**IAT alone (*n* = 63)**	**MT (*n* = 57)**	**Effect size (95% CI^a^)**	***P-*value^a^**
**Primary outcome- no (%)**					
90-day mRS 0-2	100 (83.3)	59 (93.7)	41 (71.9)	4.17 (1.17–14.89)	**0.028**
**Secondary outcomes**					
90-day mRS 0-1- no (%)	87 (72.5)	53 (84.1)	34 (59.6)	2.38 (0.91–6.19)	0.076
90-day mRS 0-3- no (%)	104 (86.7)	61 (96.8)	43 (75.4)	9.79 (1.89–50.60)	**0.006**
Successful recanalization- no (%)	108 (90.0)	57 (90.5)	51 (89.5)	0.30 (0.07–1.36)	0.118
Complete recanalization- no (%)	87 (72.5)	47 (74.6)	40 (70.2)	0.73 (0.29–1.87)	0.514
PTR, median (IQR), min	80 (50–110)	60 (40–80)	100 (80–157)	−61.44 (−80.05–−42.82)^b^	**<0.001**
OTR, median (IQR), min	514 (433–664)	510 (395–730)	528 (450–648)	−7.89 (−107.71–91.94)^b^	0.877
**Safety outcomes- no (%)**					
Any ICH within 48 h	13 (10.8)	2 (3.2)	11 (19.3)	0.13 (0.03–0.71)	**0.019**
Mortality within 90 days (mRS 6)	11 (9.2)	1 (1.6)	10 (17.5)	0.06 (0.01–0.52)	**0.011**

*ICH, intracranial hemorrhage; mTICI, modified thrombolysis in cerebral infarction; mRS, modified Rankin scale; IAT, intra-arterial thrombolysis; MT, mechanical thrombectomy; PTR, time from puncture to recanalization; OTR, time from onset to recanalization; OR, odds ratio; CI, confidence interval*.

a *Adjusted for the propensity score*.

b *The β-coefficients were calculated using a generalized linear model*.

*Bold values indicates statistical significance*.

## Discussion

Our study showed that in LVO patients who presented with minor stroke, the IAT alone group had better 90-day functional outcomes, less mortality within 90 days, and less ICH rate within 48 h compared to the MT group.

Several RCTs have demonstrated the benefits of MT for LVO patients with NIHSS > 6 (3). However, the safety and efficacy of IAT for LVO were still unclear. Multicenter Randomized Clinical Trial of Endovascular Treatment for Acute Ischemic Stroke in the Netherlands (MR CLEAN) (15) and Endovascular Treatment for Small Core and Proximal Occlusion Ischemic Stroke (ESCAPE) trails (16) allowed the use of IAT, but no subgroup analyses have been published so far. Kaesmacher et al. (2020) reported that IA urokinase, after failed, unsuccessful, or incomplete MT for anterior circulation LVO with NIHSS ≥ 6, was safe and improved angiographic reperfusion (17). The Chemical Optimization of Cerebral Embolectomy (CHOICE) RCT demonstrated that adjunct IA r-tPA after successful reperfusion following thrombectomy was more likely to yield an excellent neurological outcome at 90 days in patients with LVO (18).

In contrast to the two studies mentioned above, we compared the clinical and angiographic outcomes between IAT alone and MT for LVO patients with minor stroke and found that IAT was more beneficial than MT, with lower mortality within 90 days and ICH rate within 48 h. Several reasons could explain our findings: (1) High recanalization rate was achieved in the IAT alone group despite no significant difference between the two groups regarding the recanalization rate in our study. It has been confirmed by previous well-known studies that recanalization rate is an essential factor for better outcomes (3, 19, 20). (2) IAT is easier to perform than MT, and the relatively less complex procedure could result in shorter PTR. In this study, IAT could reduce almost one-third of the recanalization time (40 min), in which delayed treatment time is the predictive factor for increased disability and bleeding rate (21). Thus, reducing the recanalization time might be an important factor in improving clinical prognosis. (3) MT procedure is more invasive, and arterial wall injury is inevitable. Moreover, the longer the retriever or aspiration times, the greater the injury to the arterial wall, which might also increase the risk of bleeding (22). (4) In our study, large artery atherosclerosis (LAA) accounted for 85.0% of all patients; LAA might exacerbate the injury during thrombus retrieval (2, 23).

Interestingly, the IAT alone group had similar angiographic outcomes to MT groups despite their clinical outcomes being superior to the MT group. One possible explanation for this result is that LAA is the most common etiology of stroke in our study. The patients with LAA generally had good collateral (24) and a low clot burden (25). Thus, it was relatively easy to achieve vascular recanalization with IAT or MT. Another notable finding in the current study is the very high rate (93.7%) of 90-day mRS 0-2 in the IAT alone group, which may be because IAT not only could achieve successful recanalization but also improved the distal microcirculation reperfusion (10).

There are several limitations to our study. First, our study was not an RCT, which could lead to selection bias. Second, the small sample size may decrease the statistical power to reflect the actual effects of the study. In addition, the small sample size also resulted in inflated confidence intervals due to a large number of adjustments for the limited number of patients. This may impact the precision of the studies and warrant confirmation in larger cohorts. Third, the thrombolytic agents and dosage were not unique, which could confound our results. Finally, due to the high prevalence of LAA in our cohort, our results could not be easily extrapolated to other populations.

## Conclusions

IAT may prove safer and more effective than MT in patients with minor strokes. However, a large multicenter cohort or randomized controlled trial is urgently needed to clarify the results further. Furthermore, in addition to MT, it might also be worthwhile to explore IAT as an alternative to standard medical treatment in future trials.

## Data Availability Statement

The raw data supporting the conclusions of this article will be made available by the authors, without undue reservation.

## Ethics Statement

The studies involving human participants were reviewed and approved by the Ethics Committee of Beijing Tiantan Hospital. The patients/participants provided their written informed consent to participate in this study.

## Author Contributions

ZM supervised and performed quality control for the study. AW performed the statistical analysis. DS, XH, and R acquired the data and wrote the manuscript with input from all co-authors. All authors contributed to the article and approved the submitted version.

## Funding

This work was supported by the National Key Research and Development Program of China, Grant Number 2016YFC1301500.

## Conflict of Interest

The authors declare that the research was conducted in the absence of any commercial or financial relationships that could be construed as a potential conflict of interest.

## Publisher's Note

All claims expressed in this article are solely those of the authors and do not necessarily represent those of their affiliated organizations, or those of the publisher, the editors and the reviewers. Any product that may be evaluated in this article, or claim that may be made by its manufacturer, is not guaranteed or endorsed by the publisher.

## References

[1] Reeves M Khoury J Alwell K Moomaw C Flaherty M Woo D. Distribution of national institutes of health stroke scale in the cincinnati/northern kentucky stroke study. Stroke. (2013) 44:3211–3. 10.1161/STROKEAHA.113.002881 24003048 PMC4632977

[2] Rajajee V Kidwell C Starkman S Ovbiagele B Alger JR Villablanca P. Early mri and outcomes of untreated patients with mild or improving ischemic stroke. Neurology. (2006) 67:980–4. 10.1212/01.wnl.0000237520.88777.71 17000964

[3] Goyal M Menon BK van Zwam WH Dippel DW Mitchell PJ Demchuk AM. Endovascular thrombectomy after large-vessel ischaemic stroke: a meta-analysis of individual patient data from five randomised trials. Lancet. (2016) 387:1723–31. 10.1016/S0140-6736(16)00163-X 26898852

[4] Powers WJ Derdeyn CP Biller J Coffey CS Hoh BL Jauch EC. 2015 American heart association/American stroke association focused update of the 2013 guidelines for the early management of patients with acute ischemic stroke regarding endovascular treatment: a guideline for healthcare professionals from the American heart association/American stroke association. Stroke. (2015) 46:3020–35. 10.1161/STR.0000000000000074 26123479

[5] Turc G Bhogal P Fischer U Khatri P Lobotesis K Mazighi M. European stroke organisation (eso)- european society for minimally invasive neurological therapy (esmint) guidelines on mechanical thrombectomy in acute ischemic stroke. J Neurointerv Surg. (2019) 11:535–8. 10.1136/neurintsurg-2018-014568 31152058

[6] Powers WJ Rabinstein AA Ackerson T Adeoye OM Bambakidis NC Becker K. 2018 guidelines for the early management of patients with acute ischemic stroke: a guideline for healthcare professionals from the American heart association/american stroke association. Stroke. (2018) 49:e46–e110. 10.1161/STR.0000000000000158 29367334

[7] Goyal N Tsivgoulis G Malhotra K Ishfaq MF Pandhi A Frohler MT. Medical management vs. mechanical thrombectomy for mild strokes: an international multicenter study and systematic review and meta-analysis. JAMA Neurol. (2020) 77:16–24. 10.1001/jamaneurol.2019.3112 31545353 PMC6763987

[8] Zhao Y Song Y Guo Y Li Y Zhang Y Ma P. Endovascular thrombectomy vs. medical treatment for mild stroke patients: a systematic review and meta-analysis. J Stroke Cerebrovasc Dis. (2020) 29:105258. 10.1016/j.jstrokecerebrovasdis.2020.105258 32992178

[9] Furlan A Higashida R Wechsler L Gent M Rowley H Kase C. Intra-arterial prourokinase for acute ischemic stroke. The proact ii study: a randomized controlled trial Prolyse in acute cerebral thromboembolism. JAMA. (1999) 282:2003–11. 10.1001/jama.282.21.2003 10591382

[10] Khatri P. Intra-arterial thrombolysis to target occlusions in distal arteries and the microcirculation. JAMA. (2022) 327:821–3. 10.1001/jama.2021.25014 35143600

[11] Huo X Ma N Mo D Gao F Yang M Wang Y. Acute ischaemic stroke cooperation group of endovascular treatment (angel) registry: study protocol for a prospective, multicentre registry in china. Stroke Vasc Neurol. (2019) 4:57–60. 10.1136/svn-2018-000188 31105980 PMC6475078

[12] Barber PA Demchuk AM Zhang J Buchan AM. Validity and reliability of a quantitative computed tomography score in predicting outcome of hyperacute stroke before thrombolytic therapy. Aspects study group Alberta stroke programme early ct score. Lancet. (2000) 355:1670–4. 10.1016/S0140-6736(00)02237-6 10905241

[13] Zaidat OO Yoo AJ Khatri P Tomsick TA von Kummer R Saver JL. Recommendations on angiographic revascularization grading standards for acute ischemic stroke: a consensus statement. Stroke. (2013) 44:2650–63. 10.1161/STROKEAHA.113.001972 23920012 PMC4160883

[14] Ogawa A Mori E Minematsu K Taki W Takahashi A Nemoto S. Randomized trial of intraarterial infusion of urokinase within 6 hours of middle cerebral artery stroke: the middle cerebral artery embolism local fibrinolytic intervention trial (melt) Japan. Stroke. (2007) 38:2633–9. 10.1161/STROKEAHA.107.488551 17702958

[15] Berkhemer OA Fransen PS Beumer D van den Berg LA Lingsma HF Yoo AJ. A randomized trial of intraarterial treatment for acute ischemic stroke. N Engl J Med. (2015) 372:11–20. 10.1056/NEJMoa1411587 25517348

[16] Goyal M Demchuk AM Menon BK Eesa M Rempel JL Thornton J. Randomized assessment of rapid endovascular treatment of ischemic stroke. N Engl J Med. (2015) 372:1019–30. 10.1056/NEJMoa1414905 25671798

[17] Kaesmacher J Bellwald S Dobrocky T Meinel TR Piechowiak EI Goeldlin M. Safety and efficacy of intra-arterial urokinase after failed, unsuccessful, or incomplete mechanical thrombectomy in anterior circulation large-vessel occlusion stroke. JAMA Neurol. (2020) 77:318–26. 10.1001/jamaneurol.2019.4192 31816018 PMC6902179

[18] Renú A Millán M San Román L Blasco J Martí-Fàbregas J Terceño M. Effect of intra-arterial alteplase vs. placebo following successful thrombectomy on functional outcomes in patients with large vessel occlusion acute ischemic stroke: the choice randomized clinical trial. JAMA. (2022) 327:826–35. 10.1001/jama.2022.1645 35143603 PMC8832304

[19] Marks MP Heit JJ Lansberg MG Kemp S Christensen S Derdeyn CP. Endovascular treatment in the defuse 3 study. Stroke. (2018) 49:2000–3. 10.1161/STROKEAHA.118.022147 29986935 PMC6202142

[20] Alberts MJ Ollenschleger MD Nouh A. Dawn of a new era for stroke treatment: implications of the dawn study for acute stroke care and stroke systems of care. Circulation. (2018) 137:1767–9. 10.1161/CIRCULATIONAHA.118.033579 29348262

[21] Emberson J Lees KR Lyden P Blackwell L Albers G Bluhmki E. Effect of treatment delay, age, and stroke severity on the effects of intravenous thrombolysis with alteplase for acute ischaemic stroke: a meta-analysis of individual patient data from randomised trials. Lancet. (2014) 384:1929–35. 10.1016/S0140-6736(14)60584-5 25106063 PMC4441266

[22] Bourcier R Saleme S Labreuche J Mazighi M Fahed R Blanc R. More than three passes of stent retriever is an independent predictor of parenchymal hematoma in acute ischemic stroke. J Neurointerv Surg. (2019) 11:625–9. 10.1136/neurintsurg-2018-014380 30389897

[23] Lee JS Lee SJ Yoo JS Hong JH Kim CH Kim YW. Prognosis of acute intracranial atherosclerosis-related occlusion after endovascular treatment. J Stroke. (2018) 20:394–403. 10.5853/jos.2018.01627 30309234 PMC6186924

[24] Guglielmi V LeCouffe NE Zinkstok SM Compagne KCJ Eker R Treurniet KM. Collateral circulation and outcome in atherosclerotic versus cardioembolic cerebral large vessel occlusion. Stroke. (2019) 50:3360–8. 10.1161/STROKEAHA.119.026299 31658903 PMC7597992

[25] Kang DH Yoon W. Current opinion on endovascular therapy for emergent large vessel occlusion due to underlying intracranial atherosclerotic stenosis. Korean J Radiol. (2019) 20:739–48. 10.3348/kjr.2018.0809 30993925 PMC6470088

