# Supplementary material for: Intra-Arterial Thrombolysis Vs. Mechanical Thrombectomy in Acute Minor Ischemic Stroke Due to Large Vessel Occlusion
Source: Front Neurol. 2022 Jul 12;13:860987. doi: 10.3389/fneur.2022.860987 (PMC9315389; doi:10.3389/fneur.2022.860987)
Supplement: Supplementary file 1 [file Data_Sheet_1.PDF]

Supplementary Table 1. Baseline and outcomes characteristics of IAT alone and MT groups in anterior circulation and posterior circulation

Characteristics	Anterior circulation (n = 65)		P value	Posterior circulation (n = 55)		P value
	IAT alone (n = 35)	MT (n = 30)		IAT alone (n = 28)	MT (n = 27)	
Age - year, median (IQR)	61(52-70)	69(60-77)	**0.028**	58(49-62)	64(58-69)	**0.010**
Male sex - no.%	28(80.0)	21(70.0)	0.351	17(60.7)	23(85.2)	**0.042**
Systolic pressure - mmHg, median (IQR)	140(136-160)	140(130-163)	0.574	144(131-160)	159(140-166)	0.269
NIHSS score, median (IQR)	3(2-4)	3(2-4)	0.395	3(1-4)	2(0-4)	0.325
Current or previous smoking – no. (%)	15(42.9)	11(36.7)	0.612	9(32.1)	11(40.7)	0.508
Current or previous drinking- no.%	9(25.7)	7(23.3)	0.824	5(17.9)	6(22.2)	0.686
**Medical history - no. (%)**						
Atrial Fibrillation	1(2.9)	3(10.0)	0.328	0	0	NA
Diabetes Mellitus	5(14.3)	9(30.0)	0.124	5(17.9)	7(25.9)	0.469
Previous stroke	1(2.9)	4(13.3)	0.173	1(3.6)	2(7.4)	0.611
Hypertension	19(54.3)	18(60.0)	0.643	13(46.4)	18(66.7)	0.130
**TOAST classification - no. (%)**						
Large artery atherosclerosis	31(88.6)	22(73.3)	0.114	25(89.3)	24(88.9)	1.000
Cardiogenic	0(0)	3(10.0)	0.093	0	0	NA
Other etiology or unknown etiology	4(11.4)	5(16.7)	0.722	3(10.7)	3(11.1)	1.000
Occlusion Site - no. (%)						
ICA	17(48.6)	19(63.3)	0.233			
M1	12(34.3)	7(23.3)	0.333			
M2/3	4(11.4)	3(10.0)	0.853			
ACA	2(5.7)	1(3.3)	1.000			
PCA				7(25.0)	1(3.7)	0.051

V-BA				21(75.0)	26(96.3)	0.051
OTD time, median (IQR), min	240(180-317)	273(182-305)	0.921	299(218-474)	250(123-302)	**0.037**
DTP time, median (IQR), min	143(96-242)	130(118-177)	0.549	116(85-159)	160(128-310)	**0.006**
OTP time, median (IQR), min	420(314-700)	420(350-459)	0.502	453(348-635)	438(380-570)	0.814
**Peri-procedural antithrombotic and anticoagulant - no. (%)**						
Prior use of antiplatelet agents	10(28.6)	10(33.3)	**0.678**	3(10.7)	6(22.2)	0.295
Bridging IVT	3(8.6)	10(33.3)	**0.013**	1(3.6)	4(14.8)	0.193
Tirofiban	11(31.4)	13(43.3)	0.321	5(17.9)	15(55.6)	**0.004**
Heparin during EVT	7(20.0)	8(26.7)	0.525	4(14.3)	14(51.9)	**0.003**
**Primary outcome- no (%)**						
90-day mRS 0-2	31(88.6)	22(73.3)	0.114	28(100)	19(70.4)	**0.002**
**Secondary outcomes**						
90-day mRS 0-1- no (%)	28(80.0)	17(56.7)	**0.042**	25(89.3)	17(63.0)	**0.022**
90-day mRS 0-3- no (%)	33(94.3)	23(76.7)	0.069	28(100)	20(74.1)	**0.004**
Successful recanalization- no (%)	33(94.3)	28*93.3)	1.000	24(85.7)	23(85.2)	1.000
Complete recanalization- no (%)	25(71.4)	20(66.7)	0.678	22(78.6)	20(74.1)	0.695
PTR time, median (IQR), min	55(40-78)	103(70-133)	**<0.001**	70(50-95)	92(85-160)	**0.001**
OTR time, median (IQR), min	480(378-740)	495(411-639)	0.984	517(433-713)	550(470-665)	0.501
**Safety outcomes- no (%)**						
Any ICH within 48 h	2(5.7)	8(26.7)	**0.035**	0(0)	3(11.1)	0.111
Mortality (mRS 6) within 90 days	1(2.9)	7(23.3)	**0.020**	0(0)	3(11.1)	0.111

Abbreviation: MT,mechanical thrombectomy; SD, standard deviation; SBP, systolic blood pressure; IQR, interquartile range; NIHSS, National Institutes of Health Stroke Scale score; TOAST, trial of ORG 10172 in acute stroke treatment; ICA,internal carotid artery; M1, middle cerebral artery M1 segment; M2/3, middle cerebral artery M2/3 segment; ACA, anterior cerebral artery; PCA, posterior cerebral artery; V-BA, vertebrobasilar artery; OTD, time from onset to door; DTP, time from door to puncture; PTR, time form puncture to recanalization; OTP, time from onset to puncture; OTR, time form onset to recanalization; IVT, intravenous thormbolysis; IAT, intra-arterial thrombolysis.

Supplementary Table 2. Treatment effects on the 90-day mRS 0-1 according to the exploratory subgroup stratified by anterior circulation and posterior circulation

**Subgroup**	**No. of patients (n = 120)**	**OR (95% CI)**	**P for interaction**
**Infarct location**			0.587
Anterior circulation	65	1.92(0.47-7.84)	
Posterior circulation	55	3.19(0.58-17.55)	

Abbreviation: OR, odd ratio; CI, confidence interval.

Supplementary Figure 1. Distribution of modified Rankin Scale (mRS) scores at 3 months between IAT alone and MT group in the anterior circulation

Abbreviation: MT, mechanical thrombectomy; IAT, intra-arterial thrombolysis; NIHSS, National Institutes of Health Stroke Scale score; mRS, modified rankin scale.

Supplementary Figure 2. Distribution of modified Rankin Scale (mRS) scores at 3 months between IAT alone and MT group in the posterior circulation

Abbreviation: MT, mechanical thrombectomy; IAT, intra-arterial thrombolysis; NIHSS, National Institutes of Health Stroke Scale score; mRS, modified rankin scale.
