# Supplementary figures and images for: Intra-Arterial Thrombolysis Vs. Mechanical Thrombectomy in Acute Minor Ischemic Stroke Due to Large Vessel Occlusion
Source: Front Neurol. 2022 Jul 12;13:860987. doi: 10.3389/fneur.2022.860987 (PMC9315389; doi:10.3389/fneur.2022.860987)

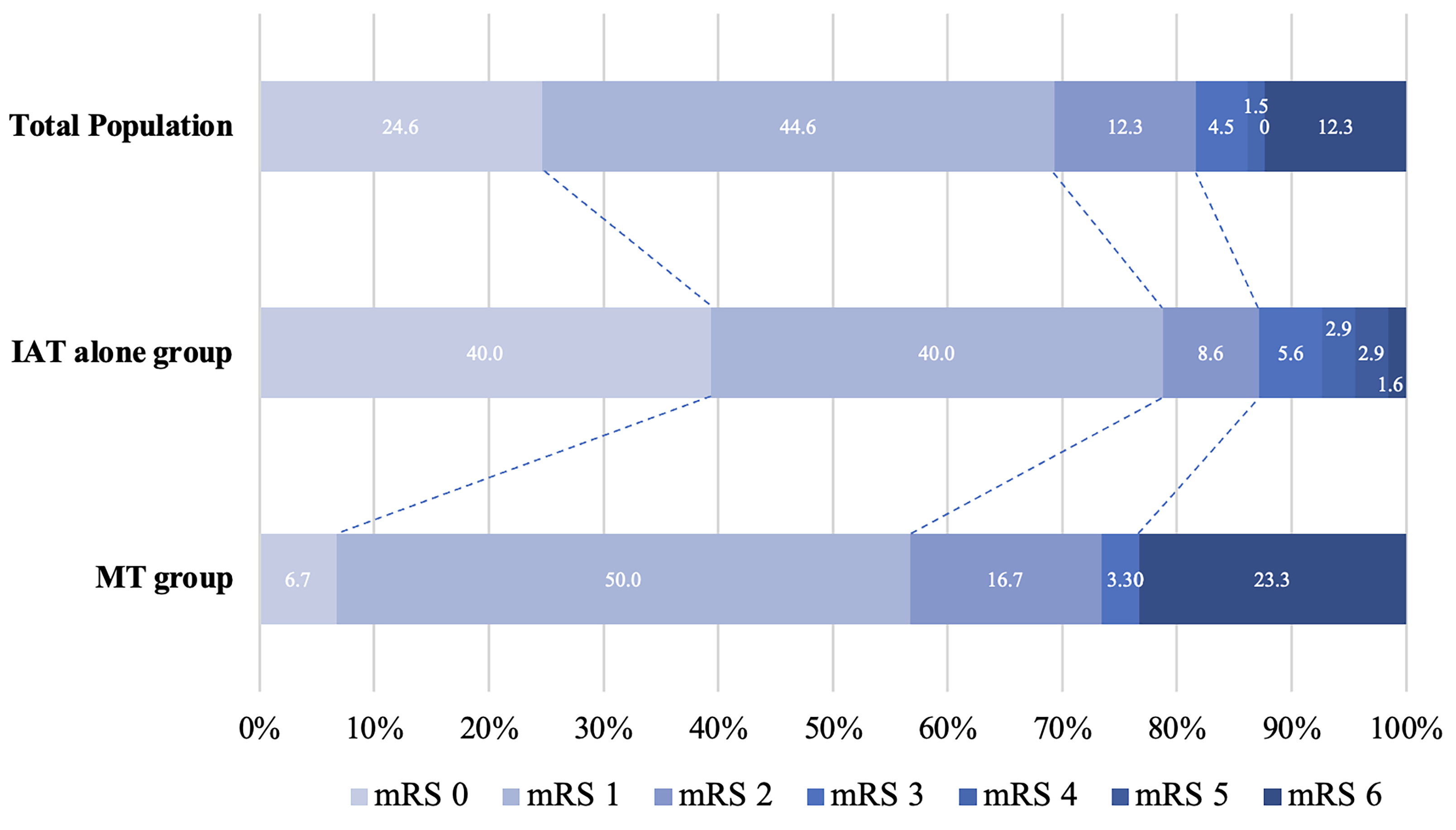

Supplement: Supplementary file 2 [file Image_1.TIF]

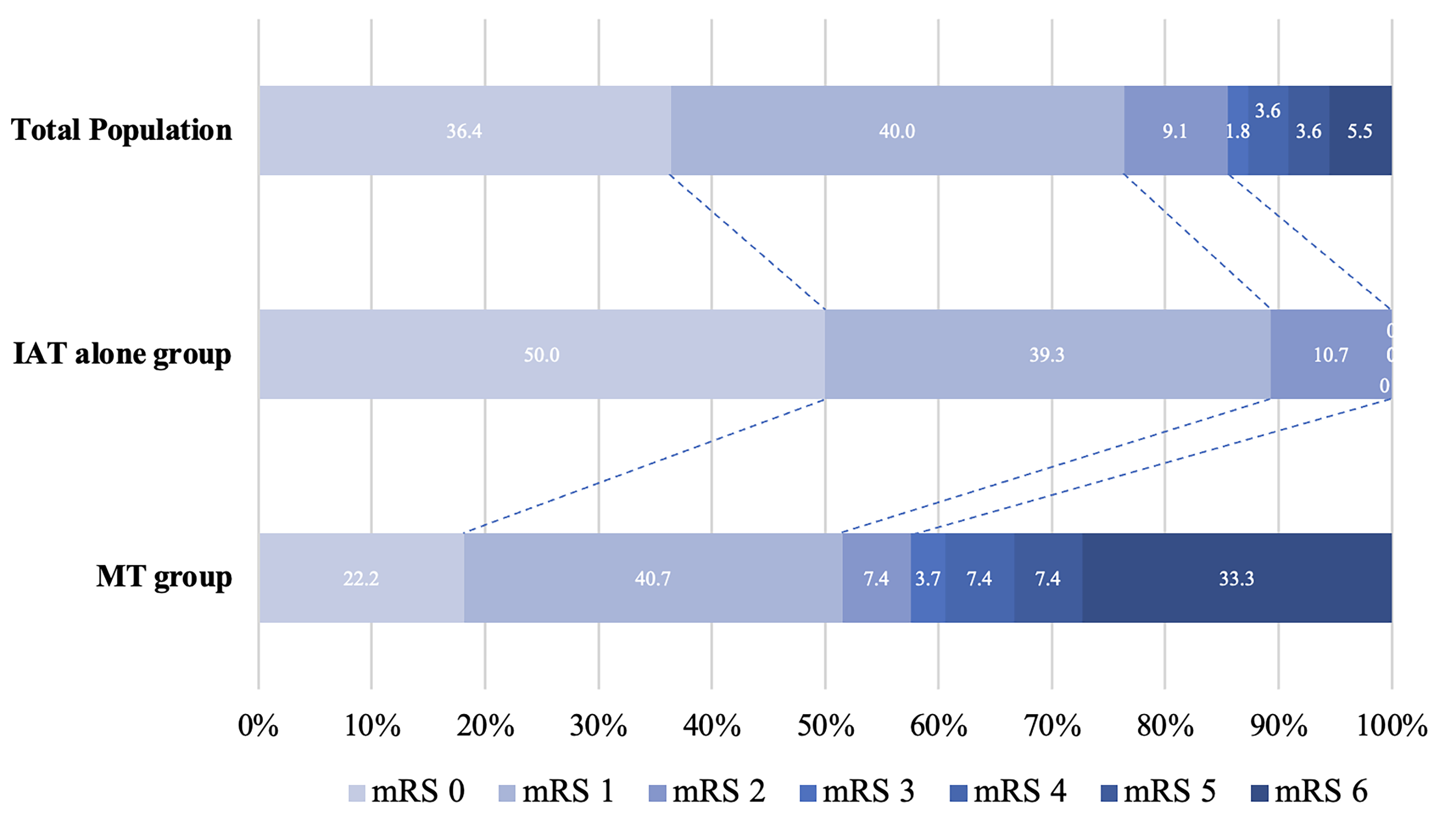

Supplement: Supplementary file 3 [file Image_2.TIF]
